# Mediterranean Diet Adherence, Sleep Disturbances and Emotional Well-Being in Skin Ulcer Burden: Insights from a Monocentric Registry

**DOI:** 10.3390/nu17213402

**Published:** 2025-10-29

**Authors:** Tonia Samela, Giulia Raimondi, Damiano Abeni, Maria Beatrice Pupa, Maria Chiara Collina, Teresa Odorisio, Alessia Paganelli

**Affiliations:** 1 Clinical Epidemiology Unit, Istituto Dermopatico Dell’Immacolata, IDI-IRCCS, 00167 Rome, Italy; t.samela@idi.it (T.S.); d.abeni@idi.it (D.A.); m.pupa@idi.it (M.B.P.); 2 Clinical Psychology Unit, Istituto Dermopatico Dell’Immacolata, IDI-IRCCS, 00167 Rome, Italy; 3 Diabetic Foot Outpatient Clinic, Istituto Dermopatico Dell’Immacolata, IDI-IRCCS, 00167 Rome, Italy; m.collina@idi.it; 4 Laboratory of Molecular and Cell Biology, Istituto Dermopatico Dell’Immacolata, IDI-IRCCS, 00167 Rome, Italy; t.odorisio@idi.it; 5 Center for Regenerative Medicine and Immunodermatology (CEDRI), Istituto Dermopatico Dell’Immacolata, IDI-IRCCS, 00167 Rome, Italy; a.paganelli@idi.it

**Keywords:** chronic skin ulcers, Mediterranean diet, patient-reported outcomes, gender differences, psychodermatology, wound healing

## Abstract

Background: Chronic skin ulcers are characterized by an impaired and delayed wound healing process, posing a major economic and healthcare burden. These multifactorial conditions are influenced by both biological, clinical and psychosocial factors. The aim of our cross-sectional study was to investigate the influence of psychosocial and lifestyle factors—specifically adherence to the Mediterranean diet, emotional health, sleep quality and demographic characteristics—on physical symptoms and clinical severity in patients with skin ulcers, using a multidisciplinary approach to identify key predictors of disease burden. Methods: A cross-sectional analysis was conducted on patients with skin ulcers, using data from a monocentric pathology registry. Collected variables included gender, age, dietary habits (specifically, adherence to the Mediterranean diet), sleep disturbances, educational level, anxiety and depressive symptoms, Physician Global Assessment (PGA), Patient Global Assessment (PtGA), and Skindex-17 (a dermatology-specific quality of life measure). Hierarchic multivariate linear regression models were applied to identify predictors of physical symptoms and clinical severity, while simultaneously controlling for potential confounders. Results: Older age, poorer adherence to the Mediterranean diet, and elevated anxiety levels emerged as the strongest predictors of worse physical symptoms, as measured by the Skindex-17. Male sex and more severe depressive symptoms were significantly associated with higher PGA scores. Our data also suggest older age and poorer Mediterranean diet adherence to influence clinical severity. Lastly, sleep disturbances were also found to correlate with patient-reported severity. Conclusions: Our study underscores the impact of psychosocial and behavioral/lifestyle factors on the clinical burden of skin ulcers through a comprehensive multidisciplinary approach. In particular, our data indicate that dietary patterns and emotional health appear to shape both symptom perception and clinical evaluation, emphasizing the need for holistic management strategies.

## 1. Introduction

Chronic skin ulcers encompass a wide range of possible etiologies, including venous insufficiency, arterial disease, pressure-related ischemia, and diabetic neuropathy. These lesions constitute a persistent challenge in wound management, not only imposing a heavy financial burden on healthcare systems worldwide but also severely impairing patients’ functional status and psychosocial well-being [1,2]. In the United States alone, the annual cost of managing chronic wounds exceeds $25 billion, reflecting the extensive use of outpatient care, long-term wound management supplies, hospitalizations, and productivity losses [1]. In fact, these wounds tend to persist for months or even years, with high rates of recurrence and complications, including infection and amputation. Moreover, the caregiver burden associated with chronic wound care extends the impact of these conditions beyond the individual, affecting family dynamics and social support systems [3].

While extensive research has focused on the biomedical mechanisms underlying chronic wound pathophysiology, a growing body of evidence suggests that psychosocial, behavioral, and demographic factors may also play critical roles in shaping healing outcomes [4,5]. This underscores the need to consider patients’ subjective symptom experiences and overall quality of life (QoL) during clinical assessment, as a patient-centered approach is more effective for addressing these complex, individualized issues than a disease-centered model focused solely on objective clinical findings. Pain, for example, is a frequently reported symptom in the chronic-ulcer population, contributing to sleep disturbances, mobility limitations, and depressive symptoms [6,7]. Nutritional status is another modifiable determinant that is often overlooked. The Mediterranean diet, rich in antioxidants, fibers, unsaturated fats, and anti-inflammatory compounds, has shown promising benefits in wound healing, especially in individuals with vascular and diabetic ulcers [8,9,10]. However, studies systematically integrating lifestyle, dietary, and psychological factors into predictive models of clinical severity and symptom burden in patients with skin ulcers are currently underrepresented in scientific literature.

By examining both clinician-rated outcomes and subjective symptom experiences, this research aims to provide a more comprehensive understanding of the biopsychosocial dimensions of chronic ulcer care, with the ultimate goal of informing personalized, multidisciplinary management strategies. To this end, we explored sociodemographic, lifestyle, and psychological correlates of clinical and patient-reported severity, using real-world data from a monocentric wound care registry.

## 2. Materials and Methods

### 2.1. Study Design

This cross-sectional, observational study was performed at IDI-IRCCS, Istituto Dermopatico dell’Immacolata Resesearch Institute (Rome, Italy). The study protocol was approved by the local Ethical Committee (Prot.N. 713/1, 1 March 2023). Research procedures were conducted in compliance with the principles of the Helsinki Declaration, with written informed consent being provided by all patients. Data were anonymized by assigning an alpha-numerical code to each enrolled subject.

Patients affected by lower-limb chronic ulcers followed up at the Skin Ulcer and Diabetic Foot Unit of IDI-IRCCS were consecutively enrolled between June 2024 and May 2025. Data collection was performed at baseline and, whenever available, at subsequent follow-up visits.

### 2.2. Study Participants

Participants’ inclusion criteria were (i) aged 18 or more of both sexes; (ii) affected by lower limb cutaneous ulcers; (iii) cutaneous ulcer duration ≥ 6 weeks; (iv) etiology of the ulcer: venous, arterial, lymphatic, mixed, pressure, diabetic. Conversely, individuals were excluded if (i) unable to provide written informed consent to participate in the study; (ii) with a confirmed diagnosis of a major psychiatric disorder (including Schizophrenia Spectrum Disorder, Bipolar Disorder, or Major Depression) or a major neurocognitive disorder (iii) atypical ulcer location (trunk, upper limbs, head/neck area); (iv) rarer etiologies not listed in the inclusion criteria (e.g., leishmaniasis, mycobacterial infections, vasculitis, pyoderma gangrenosum, etc.).

### 2.3. Collected Data and Questionnaires

At baseline, after clinical evaluation, all participants were administered case report forms for the assessment of socio-demographic and health information: gender; age; weight and height; educational level; use of tobacco; alcohol; medication intake in the last six months; sleeping disturbances (insomnia, difficulty falling asleep, nocturnal or early awakening causing deterioration in everyday activities/QoL).

A standardized clinical assessment was also carried out for each patient by treating physicians, utilizing the Physician Global Assessment (PGA) [11,12]. Given the multidisciplinary nature of our Skin Ulcer Outpatient Service, PGA was always agreed between two clinicians, one of which was invariably a dermatologist with specific expertise on chronic wounds. Aside from PGA, all the other data collection tools employed in the present study were self-administered.

An adapted version of DASS-21 [13] was used to assess emotional distress (anxiety, depression, stress), with 21 items being rated on a four-point Likert scale (from 0 = did not apply to me at all, to 3 = applied to me very much or most of the time). The General Distress (GD) score is calculated as the sum score of all the 21 items, with greater scores being related to a higher level of distress. The Italian version of Brief Illness Perception Questionnaire (Brief IPQ) [14] was used to assess how individuals tend to perceive and accommodate the emotional and practical consequences associated with their illness. The questionnaire comprises 9 items, rated on a Likert scale (0–10), all of which contribute to a total score. Higher scores indicate poorer patient adjustment to the illness.

The Skindex-17 [15] is a validated tool for measuring the impact of skin disease on patients’ lives [16], as it captures symptom burden and emotional effects experienced during the week preceding questionnaire completion on a three-step Likert scale (never; rarely/sometimes; often/always), for a total of 17 items. The Skindex-17 has two sub-scales: a first one measuring the impact on patients’ QoL (with values ≥ 50 indicating a severe impact of dermatological symptoms on patients’ quality of life), and a second sub-scale investigating experienced psychosocial distress. Scores on the psychosocial sub-scale can be categorized into three levels of severity: mild (≤20.82), moderate (20.83–37.50), and severe (≥37.51).

The Patient Global Assessment (PtGA) is a single-item measure used in this study to capture the patient’s overall perception of their health condition; it is structured as a 5-point Likert scale, and translates the patient’s subjective experience into a quantifiable score, from the best to the worst possible state, referring to disease activity [17].

Lastly, the Questionnaire for Adherence to the Mediterranean Diet (QueMD) is an instrument specifically designed for the Italian population, aimed at assessing, through a “food frequency” approach, the degree of individuals’ adherence to Mediterranean dietary patterns [18]. The scoring method provides a continuous score from 0 to 9, where “0” indicates low adherence and “9” indicates maximal adherence to the Mediterranean diet. Therefore, if the frequency of use or the type of food selected by the subject does not correspond to the rules dictated by the Mediterranean diet, a score of 0 is assigned.

### 2.4. Statistical Analysis

Descriptive statistics were computed for all variables of interest. Specifically, means (SD) and percentages were calculated for continuous and categorical variables, respectively. Hierarchical multiple linear regression analyses were conducted to examine the predictors of both Physician Global Assessment (PGA) and Patient Global Assessment (PtGA), and Skindex-17 physical and psychosocial symptoms subscales scores. For all outcomes, predictors were entered in successive blocks to evaluate their incremental explanatory power while controlling for potential confounders. In the first block, demographic variables (i.e., sex, age, and educational level) were included. In the second block, Body Mass Index (BMI) and adherence to the Mediterranean diet, were added. In the third block, psychological variables (i.e., symptoms of depression, anxiety and stress; subscales from the DASS-21) were entered, as well as the presence of sleep disturbances. In the final block, illness perception total score, and both Skindex-17 subscales were added to assess their contribution to clinician-rated severity. For each predictor, standardized beta coefficients (β), 95% confidence intervals, and *p*-values (<0.05) were reported. All statistical analyses were performed using the Statistical Package for the Social Sciences, IBM SPSS 26 [19].

## 3. Results

A total of 150 patients with lower-limb chronic ulcers were consecutively enrolled during the study period. Since recruitment was based on all eligible patients at the Skin Ulcer and Diabetic Foot Unit of IDI-IRCCS, there was no distinction between approached and included participants. Descriptive characteristics of the sample are summarized in Table 1. Missing data were minimal, involving less than 5% of the sample (7 participants for the Mediterranean diet questionnaire, 2 for BMI, and 1 each for the Skindex-17 psychosocial and symptoms subscales). Given the very low proportion, missing values were imputed using the Maximum Likelihood estimation, which has been shown to yield unbiased parameter estimates [20].

Our data indicate that depression is a significant positive predictor of clinical severity measured with PGA (β = 0.387, *p* = 0.003, 95% CI [0.020–0.091]), indicating that higher depressive symptoms could be associated with greater clinician-rated severity. Male sex also turned out to be associated with higher PGA scores (β = −0.222, *p* = 0.011, 95% CI [−0.963–−0.130]). Despite very limited, a slight association with PGA also emerged for age (β = 0.172, *p* = 0.075, 95% CI [−0.002–0.041]), suggesting a possible trend towards higher severity scores in the elderly population. Interestingly, adherence to the Mediterranean diet almost reached significance with an inverse trend respect to PGA scores (β = −0.158, *p* = 0.063, 95% CI [−0.291–0.008]), indicating that nonadherence may be linked to worse clinical outcomes. Other predictors, including the remaining socio-demographic variables, anxiety, stress, BMI, sleep disturbances, and illness perception, were not significantly associated with PGA in the final model (see Table 2 for detailed information on all predictors included in the model). Of note, the final model for PGA explained 20.4% of the variance (R^2^ = 0.204).

Other interesting results come from the assessment of patient-reported outcomes. The final model for Skindex-17 physical symptoms explained 30.4% of the variance (R^2^ = 0.304). Age turned out to be a significant positive predictor (β = 0.236, *p* = 0.007, 95% CI [0.142–0.900]), suggesting that older age could be associated with a greater physical symptom burden. Not surprisingly, psychological variables were also found to play a role in patient-reported outcomes. In fact, the presence of anxiety-related symptoms was significant (β = 0.272, *p* = 0.007, 95% CI [0.274–1.667]), suggesting that higher anxiety levels were associated with worse physical symptoms. Even more importantly, adherence to the Mediterranean diet emerged as a significant (negative) predictor of Skindex-17 scores (β = −0.220, *p* = 0.004, 95% CI [−6.433–−1.217]), with lower adherence rates being linked to greater physical symptom burden. No other variables, including the remaining socio-demographic variables, depression, stress, BMI, sleep disturbances, and illness perception, showed significant associations with Skindex-17 physical symptoms in the final model (see Table 3 for detailed information on all predictors included in the model).

Our findings also indicate that several psychological variables significantly predicted patient-reported severity, as measured by PtGA. Sleep disturbances emerged as a positive predictor (β = 0.176, *p* = 0.037, 95% CI [0.029, 0.919]), highlighting the contribution of sleep problems to worse patient-reported outcomes. Stress was also significantly correlated with PtGA (β = 0.281, *p* = 0.028, 95% CI [0.004, 0.074]), indicating that higher stress was associated with greater perceived severity. Conversely, patients reporting higher anxiety rated their condition as less severe (β = −0.242, *p* = 0.018, 95% CI [−0.087, −0.008]). Finally, the psychosocial subscale of the Skindex-17 showed a strong positive association with PtGA (β = 0.332, *p* = 0.004, 95% CI [0.005, 0.027]), suggesting that psychosocial burden plays a key role in shaping patient perception of disease severity (see Table 4). Overall, the final model accounted for 34.8% of the variance in PtGA (R^2^ = 0.348), indicating moderate explanatory power.

Lastly, in the hierarchical regression predicting Skindex-17 psychosocial symptoms, depressive symptoms emerged as strong positive predictors (β = 0.350, *p* = 0.001, 95% CI [0.471, 1.752]). Similarly, stress was significantly associated with worse psychosocial outcomes (β = 0.302, *p* = 0.009, 95% CI [0.218, 1.516]). None of the other sociodemographic or clinical variables were significantly associated with psychosocial symptoms in the final model (Table 5). Overall, this model accounted for 42.1% of the variance in Skindex-17 psychosocial scores (R^2^ = 0.421), indicating psychological distress, particularly depression and stress, to be crucial in patient perceived impact of skin disease.

## 4. Discussion

The findings of our study offer valuable insights into the psychological and lifestyle factors influencing both clinician-rated severity and patient-reported outcomes.

One of the most interesting findings is represented by the demonstration that low adherence to the Mediterranean diet is associated with worse outcomes across both clinical severity (measured with PGA) and Skindex-17 physical symptoms, pointing to a possible target for intervention. However, also depression and anxiety emerged as significant predictors, alongside age and sex. While individual lifestyle, dietary, or psychological factors have been previously associated with skin ulcer outcomes, few studies have adopted a holistic analytical framework; by integrating these variables into a single predictive model. The evidence of older age and lower adherence to the Mediterranean diet being associated with worse symptomatology (Skindex-17) is consistent with previous research suggesting that both biological aging and diet quality impact on tissue regeneration, inflammation, and metabolic balance [8,9,10]. In particular, the Mediterranean diet’s richness in polyphenols, omega-3 fatty acids, and vitamins may support endothelial function and reduce oxidative stress, potentially mitigating ulcer progression.

The prominent role of anxiety and depression as predictors of both subjective and clinician-rated disease severity aligns with existing literature on the reciprocal relationship between chronic pain, psychological distress, and wound healing [4,6]. Psychological distress can alter immune function, elevate cortisol levels, and reduce patient motivation for self-care, all of which can compromise healing and lead to adverse clinical outcomes. Furthermore, background pain, a common but under-assessed symptom in chronic ulcers, can exacerbate emotional distress and reduce sleep quality, creating a vicious cycle that hinders recovery [7]. Interestingly, PGA was significantly associated with male sex and depressive symptoms, but other factors such as age and diet only approached statistical significance. This may reflect differences in clinical and subjective thresholds for evaluating ulcer severity. Clinicians, in fact, may focus on objective parameters (e.g., ulcer size, depth, exudate), while patients experience pain, discomfort, and social limitations that are not always externally visible. The integration of patient-reported outcomes like the Skindex-17 helps bridge this gap and offers a fuller picture of disease impact [21]. The importance of psychological wellbeing in the setting of wound care is also supported by several studies showing that depression is associated slower healing, higher rates of infection, and more frequent complications across various wound types [22,23,24,25].

The association between sleeping disturbances and higher PtGA scores underscores the role of sleep as a mediator between chronic illness and well-being, pointing to the potential benefit of integrating sleep hygiene interventions into wound care. The inverse association between anxiety symptoms and PtGA, although counterintuitive, might reflect differential coping strategies, where less anxious individuals focus more on the physical consequences of the ulcer and thus report greater perceived severity.

The present study broadens our understanding of possible contributors to clinical severity and symptom burden. Our data reveal a multifaceted relationship between demographic, psychological, and lifestyle variables and the burden of chronic skin ulcers, underscoring the importance of a biopsychosocial model in wound care, echoing earlier research highlighting the importance of multidisciplinary teams that incorporate nutritional counseling, mental health services, and holistic patient care [9,26]. As the literature shows, such integrated care can improve both healing rates and patient satisfaction, especially in cases where standard debridement, compression therapy, or surgical interventions fall short.

A major strength of this study lies in its comprehensive approach: although similar variables have been individually explored in previous research, this is one of the few investigations to prospectively assess them simultaneously, allowing for a more integrated understanding of their relative contributions.

Despite the study’s strengths, especially its use of multivariate models and real-world registry data, we acknowledge some limitations of our results. First, the monocentric nature may constrain the generalizability to other populations or care settings. Despite the exclusion of subjects reporting the presence of psychiatric diagnosis, we cannot totally exclude patients with chronic skin ulcers also displaying misdiagnosed psychiatric symptomatology. Lack of screening for the possible presence of other eating disorders may impact on Mediterranean diet adherence. Additionally, self-reported measures may be influenced by social desirability and other response biases, potentially affecting the accuracy of individuals’ health status reporting. Moreover, we acknowledge that no standardized scale applicable to all types of cutaneous ulcers was planned according to our study protocol. As such our measurements lack ulcer-specific parameters, relying on PGA alone as an objective measure of disease severity. Despite ulcer-specific parameters such as wound size, depth, borders, necrosis, exudate or perilesional erythema were evaluated in course of clinical evaluation, the lack of uniformity in data collection did not allow us to perform further statistical analysis on these data. Finally, the cross-sectional design of the study limits the ability to infer causality between the considered variables. Future prospective studies with longitudinal follow-up are needed to validate these associations and assess the effects of targeted interventions.

## 5. Conclusions

This study highlights the significant role of psychosocial and lifestyle factors (such as anxiety, depression, age, and adherence to the Mediterranean diet) in shaping both patient-reported symptoms and clinician-assessed severity of chronic skin ulcers. These findings support a shift toward holistic, patient-centered care models that integrate psychological support and nutritional guidance alongside conventional wound treatment. Addressing subjective symptoms and individual experiences is essential for improving quality of life and clinical outcomes in this vulnerable population. Future research should explore longitudinal interventions targeting these modifiable factors to enhance healing trajectories and reduce the overall burden of chronic ulcers.

## Figures and Tables

**Table 1:** Descriptive statistics of the sample.

Variables	N	%
**Sex**		
Male	85	56.70%
Female	65	43.30%
**Educational attainment**		
None	5	3.30%
Elementary school	40	26.70%
Middle school	55	36.70%
High school	36	24.00%
Bachelor’s degree or higher	14	9.30%
**Marital status**		
Single	11	7.30%
Married or living together	13	8.70%
Divorced	82	54.70%
Widowed	43	28.70%
**Body Mass Index**		
BMI < 18.5 kg/m^2^	5	3.30%
BMI between 18.50 and 24.99 kg/m^2^	34	22.70%
BMI between 25 and 29.99 kg/m^2^	53	35.30%
BMI ≥ 30 kg/m^2^	56	37.30%
**Smoking**		
No	71	47.30%
Yes	19	12.70%
Former smoker	60	40%
**Alcohol use**		
No	71	51.30%
Yes	19	48.70%
**Sleep disturbances**		
No	88	58.70%
Yes	59	39.30%
	**Mean**	**(±SD)**
Age	73.52	(±11.19)
Body Mass Index	28.87	(±6.70)
Skindex-17—Physical symptoms subscale	46.37	(±23.29)
Skindex-17—Psychosocial issue-subscale	39.43	(±26.74)
Depressive symptoms	8.99	(±8.56)
Anxiety symptoms	6.99	(±6.59)
Stress	12.15	(±9.66)
Adherence to the Mediterranean diet (QueMD)	2.29	(±1.36)
Illness Perception Questionnaire (Brief IPQ)	59.95	(±110.04)

N = Number; % = Percentage; SD = Standard Deviation; BMI = Body Mass Index.

**Table 2:** Hierarchical multiple regression predicting Physician Global Assessment (PGA).

Predictor	β	*p*-Value	95% CI
Biological sex	−0.222	0.011	[−0.963, −0.130]
Age	0.172	0.075	[−0.002, 0.041]
Educational level	0.037	0.681	[−0.170, 0.259]
BMI	0.098	0.266	[−0.014, 0.049]
Adherence to the Mediterranean diet	−0.158	0.063	[−0.291, 0.008]
Depressive symptoms	0.387	0.003	[0.020, 0.091]
Anxiety symptoms	−0.125	0.263	[−0.063, 0.017]
Stress	−0.153	0.276	[−0.055, 0.016]
Sleep disturbances	−0.028	0.765	[−0.524, 0.386]
Illness perception	−0.041	0.622	[−0.002, 0.001]
Skindex-17—Physical symptoms subscale	−0.162	0.159	[−0.020, 0.003]
Skindex-17—Psychosocial issue-subscale	0.157	0.212	[−0.004, 0.018]
R^2^	0.204		

β = Beta values; 95% CI = 95% Confidence Intervals; R^2^ = R square; BMI = Body Mass Index.

**Table 3:** Hierarchical multiple regression predicting Skindex-17 Physical symptoms.

Predictor	β	*p*-Value	95% CI
Biological sex	0.104	0.190	[−2.495, 12.466]
Age	0.236	0.007	[0.142, 0.900]
Educational level	0.068	0.409	[−2.245, 5.478]
BMI	0.122	0.133	[−0.132, 0.993]
Adherence to the Mediterranean diet	−0.220	0.004	[−6.433, −1.217]
Depressive symptoms	0.153	0.172	[−0.189, 1.043]
Anxiety symptoms	0.272	0.007	[0.274, 1.667]
Stress	0.070	0.579	[−0.449, 0.800]
Sleep disturbances	0.087	0.310	[−3.967, 12.385]
Illness perception	0.018	0.821	[−0.028, 0.036]
R^2^	0.304		

β = Beta values; 95% CI = 95% Confidence Intervals; R^2^ = R square; BMI = Body Mass Index.

**Table 4:** Hierarchical multiple regression predicting Patient Global Assessment (PtGA).

Predictor	β	*p*-Value	95% CI
Sex	−0.084	0.280	[−0.184, −0.631]
Age	−0.010	0.910	[−0.022, 0.020]
Educational level	0.004	0.963	[−0.205, 0.214]
BMI	0.016	0.841	[−0.028, 0.034]
Adherence to the Mediterranean diet	−0.002	0.982	[−0.148, 0.144]
Depressive symptoms	0.042	0.713	[−0.041, 0.028]
Anxiety symptoms	−0.242	0.018	[−0.087, −0.008]
Stress	0.281	0.028	[0.004, 0.074]
Sleep disturbances	0.176	0.037	[0.029, 0.919]
Illness perception	−0.041	0.592	[−0.002, 0.001]
Skindex-17—Physical symptoms subscale	−0.107	0.303	[−0.005, 0.017]
Skindex-17—Psychosocial issue-subscale	0.332	0.004	[0.005, 0.027]
R^2^	0.348		

β = Beta values; 95% CI = 95% Confidence Intervals; R^2^ = R square; BMI = Body Mass Index.

**Table 5:** Hierarchical multiple regression predicting Skindex-17 Psychosocial symptoms.

Predictor	β	*p*-Value	95% CI
Sex	0.041	0.574	[−5.561, 9.993]
Age	0.116	0.143	[−0.100, 0.687]
Educational level	0.029	0.701	[−3.233, 4.796]
BMI	0.017	0.820	[−0.517, 0.652]
Adherence to the Mediterranean diet	−0.070	0.315	[−4.093, 1.330]
Depressive symptoms	0.350	0.001	[0.471, 1.752]
Anxiety symptoms	−0.006	0.950	[−0.747, 0.701]
Stress	0.302	0.009	[0.218, 1.516]
Sleep disturbances	0.095	0.223	[−3.242, 13.759]
Illness perception	−0.020	0.783	[−0.038, 0.029]
**R^2^**	0.421		

β = Beta values; 95% CI = 95% Confidence Intervals; R^2^ = R square; BMI = Body Mass Index.

## Data Availability

The data that support the findings of this study are available from the corresponding author upon reasonable request.
